# Glial MARK2 Modulate Inflammatory Signaling and Protects Against Tau‐induced Neurodegeneration

**DOI:** 10.1002/alz70861_108155

**Published:** 2025-12-23

**Authors:** Aoi Fukuchi, TARO SAITO, KANAE ANDO

**Affiliations:** ^1^ Tokyo Metropolitan University, Hachioji, Tokyo Japan

## Abstract

**Background:**

Neuroinflammation and glial excessive activation is a pathological hallmark of AD and related neurodegenerative diseases, while signaling mechanisms underlying this overactivation remain unclear. Microtubule affinity‐regulating kinase 2 (MARK2) has been implicated in both immune responses and AD pathology. However, the roles of MARK2 in glial‐mediated immune responses and its impact on neurodegeneration have not been fully elucidated.

**Method:**

This study employed cultured cells (BV2 murine microglial cell line), PS19 tauopathy model mice and *Drosophila* models to investigate the role of MARK2/Par‐1 in glial cell immune regulation and its effects on neurodegeneration. MARK2 expression was manipulated through knockdown or overexpression strategies. In BV2 cells, cytokine expression was assessed following stimulation with immune pathway agonists. In *Drosophila*, human tau was expressed in the retina, and glial‐specific genetic knockdown or overexpression of *Drosophila* homolog of MARK2, Par‐1, was conducted to study glial responses and tau‐induced photoreceptor degeneration. PS19 mouse brain was subjected to immunostaining to analyze MARK2 expression in homeostatic microglia and activted microglia.

**Result:**

MARK2 knockdown in BV2 cells enhanced IL‐6 expression in response to LPS, indicating MARK2 acts as a negative regulator of IL‐6 via the TLR pathway. In PS19 mice, immunostaining revealed that MARK2 was elevated in P2RY12‐positive cells but reduced in Iba‐1 positive microglia. In *Drosophila* expressing human tau in the retina, along with loss of photoreceptor neurons, expression of antimicrobial peptides including Drosomycin was upregulated, indicating activation of immune responses mediated by the Toll pathway. Glial knockdown of Par‐1 enhanced both *Drosomycin* expression and neurodegeneration, whereas overexpression of Par‐1 in glia suppressed *Drosomycin* expression and mitigated the degradation of photoreceptor neurons. These results suggest that MARK2/Par‐1 in glia negatively regulates Toll pathway‐driven inflammation and protects against tau‐induced neurodegeneration.

**Conclusion:**

This study identifies a previously unrecognized function of MARK2/Par‐1 in inflammatory cytokine expression in glial cells. Reduced activity of MARK2 might cause excessive glial activation and exacerbate neurodegeneration. These findings provide insight into the molecular underpinnings of glial inflammation in AD and highlight MARK2 as a potential therapeutic target for modulating neuroinflammatory responses.

